# Knowledge, attitudes, and behavior regarding antibiotics, antibiotic use, and antibiotic resistance in students and health care professionals of the district of Barranquilla (Colombia): A cross-sectional survey

**DOI:** 10.1016/j.heliyon.2022.e11378

**Published:** 2022-11-07

**Authors:** S. Salcedo, L. Mora, D.A. Fernandez, A. Marín, I. Berrío, H. Mendoza-Charris, E.P. Viana-Cárdenas, M. Polo-Rodríguez, L. Muñoz-Garcia, J. Alvarez-Herrera, G. Olivares-Goenaga, Y. Jimenez-Castro, V. Castro del Portillo, S. Chiquillo-Gómez, L. Barrios-Matute, J. Villa-García, Y. Gonzalez-Mattos, J. Torres-Barraza, R. Jaraba-Coronado, R. Maestre-Serrano

**Affiliations:** a Universidad Simón Bolívar, Facultad de Ciencias de la Salud, Barranquilla, Colombia; b Hospital General de Medellín, Luz Castro Gutiérrez E.S.E., Medellín, Colombia; c Corporación Para Investigaciones Biológicas - Medical and Experimental Mycology Group, Medellín, Colombia; d Alcaldía de Barranquilla, Secretaría de Salud distrital, Barranquilla, Colombia; e Clínica Centro S.A., Barranquilla, Colombia; f Organización Clínica Bonnadona prevenir SAS, Barranquilla, Colombia; g Organización Clínica General del Norte, Barranquilla, Colombia; h Clinica La Merced, Barranquilla, Colombia; i Clínica del Caribe, Barranquilla, Colombia; j Clínica Portoazul, Barranquilla, Colombia; k Mi Red Barranquilla IPS, Barranquilla, Colombia; l Clínica La Asunción, Barranquilla, Colombia; m Clinica El Carmen, Barranquilla, Colombia; n Clínica Iberoamérica, Barranquilla, Colombia; o La Misericordia Clínica Internacional, Center for Clinical and Translational Research, Barranquilla, Colombia; p Promosalud Clínica El Prado, Barranquilla, Colombia

**Keywords:** Microbial resistance, Antibiotics, Knowledge, Attitudes and practices, Colombia

## Abstract

**Objective:**

To evaluate the knowledge, attitudes and behavior regarding antibiotics, use of antibiotics, and antibiotic resistance in students and health care professionals of the district of Barranquilla, Colombia.

**Study design:**

Descriptive, cross-sectional.

**Methods:**

A sample of 399 respondents was selected, that included health professionals and medical students from 12 health institutions in the district of Barranquilla (Colombia), using an established stratified sampling method. Each of the respondent professionals completed a survey that included 43 items in the Likert scale. A descriptive analysis of the study variables was performed using the software SPSS version 25.

**Results:**

Most of the respondents were women (64.4%), aged between 26 and 35 years (47.6%); 28.8% were nurses and 26.1% general practitioners, with ≤10 years of professional experience (63.4%). Overall, the survey revealed that the participants had considerable knowledge about antibiotic use (89.5%–98% correct answers) and the spread of antibiotic resistance (67.4%–89% correct answers). Approximately 74% of the respondents agreed or fully agreed with the questions related to the management of infections and the provision of advice.

**Conclusions:**

The present study revealed that most of the health care professionals surveyed had a good knowledge about antibiotic use, although strategies must be developed to strengthen knowledge regarding the spread of antibiotic resistance. Likewise, it is important to identify opportunities for improvement related with access to the guidelines and/or materials necessary to treat infections and to provide advice on antibiotic use and antibiotic resistance.

## 1 Introduction

Antibiotic resistance is one of the most serious public health problems worldwide [1]. The clinical impact of it, is increasing with time and it is reflected in higher infections and mortality rates mainly. By 2022 the European Union/European Economic Area (EU/EEA) showed that, each year more than 670.000 infections are due to bacteria resistant to antibiotics and approximately 33.000 people die as a direct consequence [2] It is well known that AR is being accelerated by the misuse and/or overuse of antibiotics between other potential causes. Across the time, bacteria has developed resistance to the main antibiotics turning a challenge the treatment of some infections due the lack of new medicaments [1]. For example, Colistin is consider as an important antimicrobial agent used mostly for multi-drug resistant bacteria; however, in the past decades the colistin-resistant isolates has been reported as consequence of the causes previously mentioned [3]. Systematic review made by Dadashi et al in 2021, aimed to evaluate the global prevalence and distribution of colistin resistance genes among human clinical isolates of *Escherichia coli*. They retrieved that in Asia, Europe, America, Africa and Oceania, the prevalence of mobile colistin resistance (mcr)-harbouring colistin-resistant *E. coli* was 66.72%, 25.49%, 5.19%, 2.27% and 0.32 %, respectively. These results suggested that the dissemination of this strain is worldwide arising concerns between countries as well [4]. Health care professionals play an essential role in the context of antibiotic resistance when considering the inappropriate or abusive use of these drugs. This is considerably more important when they are in charge of the administration, dispensation, and prescription of antibiotics in the clinics and hospitals where they work [5]. Therefore, this population plays a key role in the design of strategies that allow raising awareness about the proper and rational antibiotic use through communication and education.

Barranquilla City, along with its metropolitan area, concentrate the largest number of health care provider institutions in the Colombian Caribbean region, and therefore, baseline studies aimed at professionals working in these institutions are required that allow identifying intervention needs on knowledge of antibiotics, antibiotic use, and resistance, and changes in behavior related with the prescription of antibiotics and antibiotic resistance.

The present study aimed to assess the knowledge, attitudes, and behavior regarding antibiotics, antibiotic use, and antibiotic resistance in students and health care professionals from health institutions in the district of Barranquilla and its metropolitan area in Colombia.

## 2 Results

Table 1 shows the demographic characteristics of the respondents, most of whom were women (64.4%), aged 26–35 years (47.6%); furthermore, 28.8% were nurses and 26.1% general practitioners, with ≤10 years of professional experience (63.4%).

**Table 1:** Demographic characteristics of the health professionals participating in the survey.

Variables	n (%)	CI (95%)
*Sex*		
Man	125 (31.3)	26.9–36.0
Woman	257 (64.4)	59.6–69.0
I'd rather not answer	17 (4.3)	2.6–6.6
*Age (years)*		
18–25	53 (13.3)	10.2–16.9
26–35	190 (47.6)	42.7–52.5
36–45	90 (22.6)	18.7–26.9
46–55	47 (11.8)	8.9–15.2
56–65	18 (4.5)	2.8–6.9
>65	1 (0.3)	0.0–1.2
*Profession/Specialty*		
Bacteriologist/Microbiologist	14 (3.5)	2.0–5.7
General Surgeon	10 (2.5)	1.3–4.4
Emergency medical coordinator	5 (1.3)	0.5–2.8
Medical coordinator	12 (3.0)	1.6–5.1
Chief Nursing Officer	115 (28,8)	24.5–33.4
Epidemiological Surveillance Chief Nursing Officer	10 (2.5)	1.3–4.4
Epidemiologist	3 (0.8)	0.2–2.0
Medical Student/Intern	41 (10.3)	7.6–13.6
Gynecologist	9 (2.3)	1.1–4.1
Infectologist	4 (1.0)	0.3–2.4
Head of Quality Control	10 (2.5)	1.3–4.4
General Practitioner	104 (26.1)	21.9–30-5
Intensive Care Physician	12 (3.0)	1.6–5.1
Internist	19 (4.8)	3.0–7.2
Pediatrician	11 (2.8)	1.5–4.7
Pharmacist-chemist	11 (2.8)	1.5–4.7
Residents (Medical Surgical Specialty)	9 (2.3)	1.1–4.1
*Professional experience (Years)*		
0–10	253 (63.4)	58.6–68.0
>10	146 (36.6)	32.0–41.4

n: Number of respondents; %: percentage; CI (95%): 95% confidence intervals.

**Figure 1:**
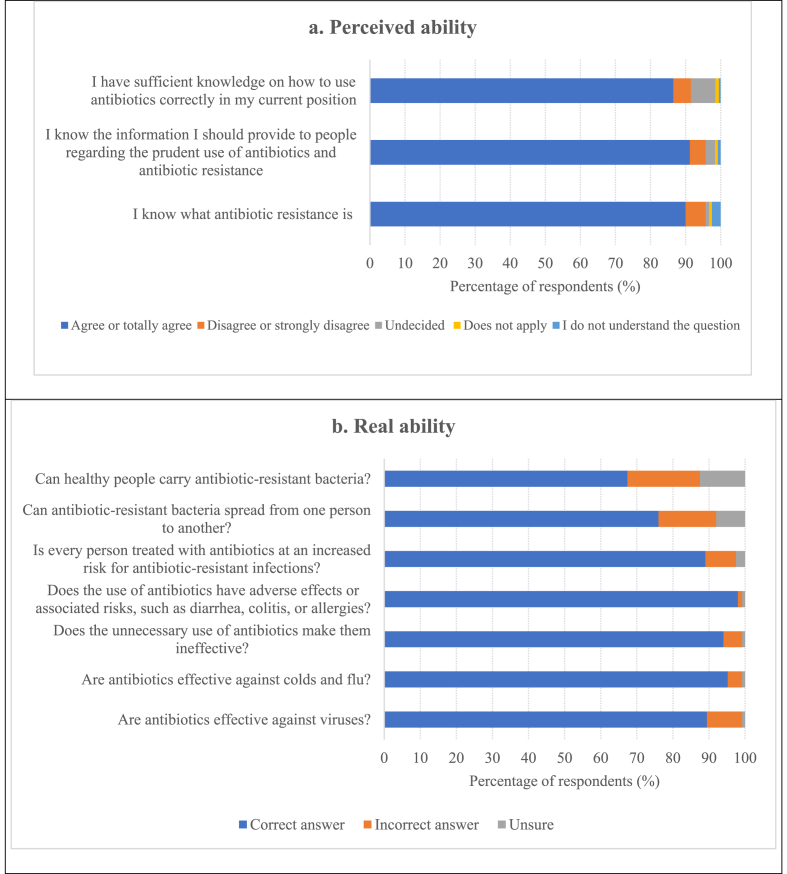
a. Percentage of respondents who agreed or disagreed with the questions related with knowledge: perceived ability (n = 399). b. Percentage of correct and incorrect answers to questions regarding knowledge: real ability.

Nearly 86.5%–91.2% of the respondents agreed or strongly agreed with the questions related with the perceived capacity (Figure 1). With respect to the seven questions formulated regarding real capacity, it was found that more than 88% of the respondents answered the questions related to antibiotic use correctly. However, the questions with the lowest percentage of correct answers were those related to the spread of antibiotic resistance (76% and 67.4%, respectively) (Figure 1).

Approximately 74% of respondents either agreed or fully agreed that they have easy access to the guidelines needed to treat infections, that they have easy access to the materials needed to provide advice on the cautious use of antibiotics and on antibiotic resistance and that they have good opportunities to offer advice to people about the cautious use of antibiotics (Table 2).

**Table 2:** Percentage of respondents who interact directly with the patient/public and who agreed or disagreed with the questions related with the management of infections and the provision of advice (n = 399).

Questions	Agree or totally agree	Disagree or strongly disagree	Undecided	Does not apply	I do not understand the question
	n (%)	n (%)	n (%)	n (%)	n (%)
Can I easily access the guidelines I need to treat infections?	296 (74.2)	33 (8.3)	38 (9.5)	28 (7.0)	4 (1.0)
Can I easily access the materials I need to provide advice on the prudent use of antibiotics and antibiotic resistance?	302 (75.7)	36 (9.0)	42 (10.5)	15 (3.8)	4 (1.0)
Do I have good opportunities to offer advice to people on the cautious use of antibiotics?	298 (74.7)	41 (10.3)	38 (9.5)	21 (5.2)	1 (0.3)

n: Number of respondents; %: percentage.

Between 82% and 86.2% of the individuals surveyed, considered that they played a key role in helping to control antibiotic resistance and that there was a connection between the prescription, dispensation or administration of antibiotics and the emergence and spread of antibiotic-resistant bacteria (Table 3).

**Table 3:** Percentage of respondents who agreed or disagreed with the questions related with motivation (n = 399).

Questions	Agree or totally agree	Disagree or strongly disagree	Undecided	Does not apply	I do not understand the question
	n (%)	n (%)	n (%)	n (%)	n (%)
Am I aware of the connection existing between my prescription or dispensation or administration of antibiotics and the emergence and spread of antibiotic-resistant bacteria?	344 (86.2)	27 (6.8)	12 (3.0)	7 (1.7)	9 (2.3)
Do I play a key role helping to control antibiotic resistance?	327 (82.0)	26 (6.5)	27 (6.7)	17 (4.3)	2 (0.5)

n: Number of respondents; %: percentage.

Among the 399 professionals surveyed, 174 (43.6%) were eligible to prescribe antibiotics; most of them were general practitioners (52.3%), internists (10.3%), intensivists (6.9%), general surgeons (5.8%), and pediatricians (5.2%). The frequency of antibiotic prescription was 55.8% on a daily basis, 36.8% weekly, and 7.5% on a monthly or quarterly basis. The daily prescription of antibiotics was mainly given by general practitioners (51.5%), followed by internists (14.4%) and intensivists (9.2%).

Regarding the decisions made when prescribing antibiotics, the support, and access to the guidelines by the prescribers, it was found that between 83.2% and 92.3% of respondents agreed or totally agreed that they felt confident when making decisions related with the prescription of antibiotics, relied on the antibiotic guidelines available, took into account antibiotic resistance when treating a patient, had easy access to the guidelines on the antibiotics needed to treat the infections, and felt supported not to prescribe antibiotics when these were not needed (Table 4). The most frequent reason for antibiotic prescriptions, even when they would have preferred not to prescribe them, during the week before the conduction of the survey, was for fear of a worsening of the patient status or for fear of complications, since 42.9% did so at least once a week (Table 5).

**Table 4:** Percentage of prescribing professionals who answered that they agreed or disagreed with the questions related with antibiotic prescription decisions, support, and accessibility to the guidelines.

Questions	Agree or totally agree	Disagree or strongly disagree	Undecided	I do not understand the question
	n (%)	n (%)	n (%)	n (%)
I feel confident making decisions about antibiotic prescription (173)	154 (89.0)	11 (6.4)	8 (4.6)	0 (0.0)
I rely on the antibiotic guidelines available to me (168)	152 (90.5)	8 (4.8)	7 (4.2)	1 (0.6)
I take antibiotic resistance into account when treating a patient (169)	156 (92.3)	7 (4.1)	6 (3.6)	0 (0.0)
I have easy access to the antibiotic guidelines I need to treat infections (171)	149 (87.1)	13 (7.6)	9 (5.3)	0 (0.0)
I feel supported not to prescribe antibiotics when they are not needed (167)	139 (83.2)	12 (7.2)	15 (9.0)	1 (0.6)

n: Number of respondents; %: percentage.

**Table 5:** Reasons for prescribing antibiotics, even when the prescriber would have preferred not to.

Question	Once a day	More than once a day	Once a week	More than once a week	Rarely	Never	I cannot remember
	n (%)	n (%)	n (%)	n (%)	n (%)	n (%)	n (%)
During the past week, how often have you prescribed antibiotics for fear of the patient's health deterioration or for fear of complications? (n:170)	14 (8.2)	6 (3.5)	35 (20.6)	18 (10.6)	46 (27.1)	46 (27.1)	5 (2.9)
During the past week, how often have you prescribed antibiotics because it took you less time to do so than to explain why they were not indicated? (n:169)	15 (8.9)	7 (4.1)	17 (10.1)	5 (2.9)	30 (17.8)	90 (53.3)	5 (2.9)
During the past week, how often have you prescribed antibiotics in situations where you were unable to follow up the patient? (n:167)	12 (7.2)	7 (4.2)	22 (13.2)	15 (8.9)	35 (21.0)	68 (40.7)	8 (4.8)
During the past week, how often have you prescribed an antibiotic to maintain the relationship with the patient? (n:167)	19 (11.3)	5 (3.0)	5 (3.0)	10 (6.0)	17 (10.2)	103 (61.7)	8 (4.8)
During the past week, how often have you prescribed antibiotics because you were unsure about the infection diagnosis? (n:167)	15 (8.9)	8 (4.8)	10 (6.0)	13 (7.8)	35 (21.0)	81 (48.5)	5 (3.0)

n: Number of respondents; %: percentage.

## 3 Discussion

There are few published studies in Colombia about the knowledge, attitudes, and behavior regarding antibiotics and antibiotic resistance in health professionals. There is a previous study that evaluated the knowledge about the prescription of antibiotics in general practitioners and specialists in three Colombian cities, including Barranquilla [6]; However, our study is different from that paper, since it assesses other issues in addition to the prescription of antibiotics and includes other health professionals, in addition to prescribers, in the study population, with chief nurses and general practitioners being the most representative professionals surveyed within the sample analyzed. It is important to highlight that in our study most of the professionals were between 26 and 35 years of age and had ≤10 years of professional experience. This could explain the results obtained regarding knowledge, since our study found that the respondents had a good perception about their knowledge about the prudent and correct use of antibiotics and antibiotic resistance. However, when assessing the real capacity and although a good knowledge about antibiotic use could be observed, knowledge regarding the spread of antibiotic resistance should be strengthened. These results about knowledge, both regarding perceived capacity and real capacity, are similar to those reported in a previous study carried out in health workers from 30 countries of the European Union [5], in which between 80 and 96% of respondents agreed or strongly agreed with the perceived ability questions, compared to our study, which found between 86.5% and 91.2% of the participants; The same thing happened with the actual ability questions, in which it was found that between 75% and 97.5% answered the questions asked correctly, compared to our study in which this percentage varied between 67.4 % and 98%, identifying in both studies the following questions with the lowest percentage of correct answers: Can antibiotic-resistant bacteria spread from one person to another? and Can healthy people carry antibiotic-resistant bacteria? Other study conducted in India found a deficient knowledge related with the correct use of antibiotics in doctors, nurses, pharmacy workers and informal health providers, finding better knowledge in doctors compared to other professionals [7, 8]. However, in another studies carried out in doctors and nurses in Canada and Nepal, it was also found that doctors have high knowledge about microbial resistance compared to nurses, especially in the older groups and work experience [8, 9].

The above could also have occurred in the present study regarding the questions about knowledge that were answered incorrectly by our respondents, as the sample population in our study was also a heterogeneous one in terms of professions, which could lead to incorrect answers, as the professionals included in the sample could have various levels of training on antibiotics, antibiotic use, and antibiotic resis-tance.

When considering our results and those from these other studies, it is important to design education and communication strategies for our population that impact the strengthening of this knowledge.

Both the ability and the opportunity to influence the motivation of an individual to generate positive behaviors [10], which could explain the high percentage of respondents who agreed or completely agreed with the motivational questions, for which they acknowledge the existence of a connection between the prescription or dispensation or administration of antibiotics and the emergence and spread of antibiotic-resistant bacteria and the key role they play in controlling antibiotic resistance. However, and in accordance with the results obtained, it is important to identify opportunities for improvement associated with the access to the guidelines and/or materials necessary to treat infections and to provide advice on antibiotic use and antibiotic resistance in order to address the barriers that could arise in the prevention and control of antibiotic resistance.

This study found that more than 80% of the prescribers surveyed agreed with the questions related with confidence when making decisions about the prescription of antibiotics, the accessibility to the guidelines on antibiotics to treat infections, and about the support felt not to prescribe antibiotics when they are not necessary. Despite the latter, reasons for an inadequate prescription of antibiotics were identified by the surveyed prescribers, the main one being fear of a worsening of the patient status or fear of complications. The least frequent reason being to maintain the relationship with the patient. Both indulgence and fear have been the most frequent attitudes found to influence the improper prescription of antibiotics in previous studies both in Colombia and other countries of the world [6, 11, 12, 13]. It is important to note that most prescribers in the present study were general practitioners with little professional experience, which could explain this type of attitudes that influence these behaviors. Furthermore, a recent study on medical students and other healthcare-related professions in Italy revealed that despite having the required knowledge, they did not apply it when making good use of antibiotics [14]. It is important to identify all such situations as they have an impact on the excessive and unnecessary use of these drugs. Therefore, the implementation of strategies that strengthen these behaviors through professional training and professional practice, becomes essential, since recent studies in countries of the European Union have identified that the prescription of antibiotics lacks the necessary relevance in medical curricula [15, 16].

This study has some limitations, because did not include all health institutions in the district of Barranquilla, as some of the institutions summoned decided not to participate and others did not meet the inclusion criteria. In the participating institutions, the largest number of physicians hired in the general hospitalization and emergency rooms are general practitioners. It is common to observe that in the daily practice of antibiotic prescription, the general practitioner prescribes under the directive of the specialist, and therefore, it was not possible to differentiate between the prescription roles of the general practitioner and the specialist physician. The data doesn't contain items regarding right antibiotic selection based on accurate diagnosis and right antibiotic dose and duration selection. Finally, are necessary future studies outside heath institutions, could help in understanding the general awareness in antibiotic and its use among people.

## 4 Conclusions

The present study revealed that most of the health care professionals surveyed had a good knowledge about antibiotic use, although strategies must be developed to strengthen knowledge regarding the spread of antibiotic resistance. Likewise, it is important to identify opportunities for improvement related with access to the guidelines and/or materials necessary to treat infections and to provide advice on antibiotic use and antibiotic resistance in order to control barriers that hinder accessibility to the guidelines and materials necessary to guide appropriate practices in the treatment of infections and in the prevention and control of antibiotic resistance.

## 5 Materials and methods

The population included in this cross-sectional study was comprised of medical students and health care professionals from 12 health care institutions in the district of Barranquilla and its metropolitan area and included health professionals and specialists who prescribe antibiotics and are members of the operational and coordination teams for surveillance and control programs for antimicrobial resistance and infections associated with health care: Physicians, specialists in internal medicine, general surgery, pediatrics, intensive medicine, infectious diseases, epidemiology, auditing, management, nursing, chemistry and pharmacy, microbiology, with a minimum of 6 months of employment at the institutions included in the study. These health institutions were selected because they had microbiology laboratories equipped with automated methods for the identification of multidrug resistance and to use the Whonet 5.6 software.

We exclude health institutions without microbiological data and health professionals who at the time of completing the survey were disabled, on maternity leave and vacations.

The sample was calculated from an approximate population size of 3,448 health care professionals linked to the participating health care institutions, with a 95% confidence interval and a power of 80% for a sample of 346 professionals, which increased by approximately 15% for a final sample size of 399 respondents. Each of these subjects was selected according to a proportionate stratified sampling, in order to maintain the proportions observed in the study population.

A survey was applied between april and may 2021; the process was carried out online using the Microsoft Office 365 forms application, on a voluntary and anonymous basis. The survey was designed and validated by a previous study and included 43 items, that included statements knowledge using a true or false answer and statements assessing attitudes and behaviours using a 5 point Likert scale: strongly agree, agree, neither agree, nor disagree, strongly disagree [5].

Demographic variables and variables related to knowledge, attitudes, and behavior regarding antibiotics, antibiotic use, and antibiotic resistance were assessed.

Descriptive statistics based on absolute and relative frequencies were used to analyze the information. All the analyses were performed in the open epi and SPSS version 25 software.

## Declarations

### Author contribution statement

Soraya Salcedo, Laura Mora, Ronald Maestre-Serrano : Conceived and designed the experiments; Analyzed and interpreted the data; Wrote the paper.

Dinno Fernandez, Adriana Marín, Indira Berrío Medina: Conceived and designed the experiments; Wrote the paper.

Humberto Mendoza Charris, Erika Paola Viana Cárdenas, Michelle Polo-Rodríguez, Lorena Muñoz-García, Juany Alvarez-Herrera, Gisselle Olivares-Goenaga, Yorleidis Jimenez-Castro, Violeta Castro del Portillo, Sylena Chiquillo-Gómez, Lidys Barrios-Matute, Jaime Villa-García, Yolima Gonzalez-Mattos, Jose Torres-Barraza, Rafael Jaraba-Coronado: Contributed reagents, materials, analysis tools or data; Wrote the paper.

### Funding statement

This work was supported by Pan American Health Organization/World Health Organization (Small Grants Scheme for Operational or Implementation Research to tackle the threat of Antimicrobial Resistance).

### Data availability statement

Data included in article/supp. material/referenced in article.

### Declaration of interest’s statement

The authors declare no conflict of interest.

### Additional information

No additional information is available for this paper.
